# Modelling Volume Change and Deformation in Food Products/Processes: An Overview

**DOI:** 10.3390/foods10040778

**Published:** 2021-04-05

**Authors:** Emmanuel Purlis, Chiara Cevoli, Angelo Fabbri

**Affiliations:** 1 CIDCA, UNLP, CONICET, La Plata 1900, Argentina; emmanuel.purlis@quimica.unlp.edu.ar; 2 Department of Agricultural and Food Sciences, Alma Mater Studiorum, Università di Bologna, 47521 Cesena, Italy; angelo.fabbri@unibo.it

**Keywords:** cellular solids, hyperelastic material, mechanical modelling, multiphysics, multiscale modelling, porosity, solid mechanics, texture, virtualization, viscoelastic material

## Abstract

Volume change and large deformation occur in different solid and semi-solid foods during processing, e.g., shrinkage of fruits and vegetables during drying and of meat during cooking, swelling of grains during hydration, and expansion of dough during baking and of snacks during extrusion and puffing. In addition, food is broken down during oral processing. Such phenomena are the result of complex and dynamic relationships between composition and structure of foods, and driving forces established by processes and operating conditions. In particular, water plays a key role as plasticizer, strongly influencing the state of amorphous materials via the glass transition and, thus, their mechanical properties. Therefore, it is important to improve the understanding about these complex phenomena and to develop useful prediction tools. For this aim, different modelling approaches have been applied in the food engineering field. The objective of this article is to provide a general (non-systematic) review of recent (2005–2021) and relevant works regarding the modelling and simulation of volume change and large deformation in various food products/processes. Empirical- and physics-based models are considered, as well as different driving forces for deformation, in order to identify common bottlenecks and challenges in food engineering applications.

## 1. Introduction

In many processes involving solid and semi-solid foods, significant volume change and large deformation occur in the products. Some typical examples include the following: shrinkage of fruits and vegetables during convective drying and of meat products during cooking, dough/bread expansion during proofing and baking, expansion in extrusion and puffing to produce snacks and breakfast cereals, and swelling during soaking of pulses. In some cases, these phenomena are positive and indeed a characteristic feature of the product/process, like expansion in baking and extrusion. On the other hand, they can represent undesired changes in other situations, e.g., excessive shrinkage during drying and cooking. However, in any case, for a wide range of processes, operating conditions, and food materials, significant volume change and deformation are part of the processes and, thus, are inevitable. Therefore, there is a need for better understanding the fundamental mechanisms of these phenomena in the context of food engineering, that is, to develop scientific knowledge and useful tools to describe and predict the relationships between processing conditions and behavior of food materials. The main goal is then to steer such phenomena towards the design of food products/processes that achieve multiple objectives involving food safety and quality (nutritional and sensory), as well as process efficiency. In this sense, modelling and numerical simulation can play an important role, providing adequate frameworks and quantitative tools for a systematic and robust analysis [1].

The occurrence and development of volume change and deformation in foods during processing can be explained by considering both material properties and driving forces established by a given process and operating conditions, like in other transport phenomena. On the one hand, most of solid and semi-solid foods are recognized as very complex biomaterials [2]; they can be described either as multiphase mixed dispersed systems or as multiphase capillary–porous media with a deformable, hygroscopic, and amorphous matrix or skeleton made of biopolymers (e.g., polysaccharides and proteins), also containing small molecular species (e.g., salts and sugars) and water [3,4]. The microstructural organization of the different phases and their physical state, and the presence of water, which is the main plasticizer, largely determine the mechanical properties and rheological behavior of foods [4,5]. On the other hand, a certain process establishes the driving forces, e.g., temperature, moisture, pressure, and/or mechanical stress gradients, while operating conditions regulate their intensity. Considering the non-equilibrium or dynamic nature of food processing, the role of water as plasticizer is essential, since, together with temperature, they determine the state of amorphous materials via the glass transition (*T*_g_) concept [6]. In general, at low temperature and/or low water content, foods are in a solid-like brittle state, the so-called glassy state. However, an increase in temperature above the *T*_g_ range (or an increase in water content) produces the glass transition: Foods change their state to rubbery (liquid-like) and now behave as soft viscoelastic materials [7,8]. Another important transition is denaturation of proteins; in the case of meat cooking, denaturation of proteins induced by heat plays a key role in structural changes [9]. So, volume change and deformation in foods are the result of complex and dynamic relationships between composition and structure of foods, and driving forces given by processes and operating conditions. Different mechanisms of deformation are discussed later for typical food products/processes.

Creation and transformation of structures or structuring of materials has a significant impact on different properties of foods, i.e., physical, transport, sensory, and also nutritional properties [10]. Volume change and deformation, in addition to changes in moisture content and temperature during processing, can generate variations in porosity and (apparent) density of foods, thus modifying the transport and mechanical properties of the materials [8,11]. In this sense, texture profile analysis (TPA), which can be thought as an imitation of mastication or chewing process, is often used to relate mechanical measurements to subjective sensation (sensory attributes), thus making food texture characteristics more predictable [12]. For instance, the Young’s modulus, a mechanical property of materials, is considered an important measure or proxy for texture. A few efforts have been made to associate process conditions and transport phenomena with mechanical properties and finally texture of food products, by using this mechanical property [13,14,15]. Furthermore, structural modifications due to different processing methods and pathways certainly influence the oral processing and overall digestion process of foods [16,17,18]. Besides sensory aspects, the structure and, thus, texture of foods can influence oral processing behavior, helping to moderate eating rate and energy intake [19]. On the other hand, volume change and deformation imply the variation of food geometry and also movement of solid skeleton, and thus need to be taken into account when modelling transport processes for a correct calculation of gradients, fluxes and average values of dependent variables [20]. This represents an additional challenge for modelling and simulation of food processes, besides the availability of thermophysical properties and experimental validation of numerical predictions [21].

So far, it is evident the underlying complexity of modelling and simulating volume change and deformation of foods during processing, but at the same time, the importance and thus the need to address this intricate problem. Actually, the development of the next generation of food process models, i.e., digital twins (virtual replica of the real process), certainly requires the inclusion of the previously described aspects, towards a holistic and comprehensive approach for food product/process design, considering the current and future challenges of agri-food industry [22]. Therefore, the objective of this article is to provide a general review of the more recent (2005–2021) and relevant works that have addressed the modelling and simulation of volume change and large deformation in different food products/processes, from a food engineering perspective. That is, it is not intended to be an extensive and detailed or systematic review of all models and/or numerical methods reported in the literature, but an overview of significant contributions in different applications with focus on large deformation of solid-like materials, which can be taken as reference for further studies and developments. By taking this transversal and comprehensive perspective, we aim at condensing the common bottlenecks and challenges shared by main applications, since most of available review articles are rather focused either on a single product/process or on a specific modelling approach. The review is organized as follows: In Section 2, different modelling approaches are described upon an ad hoc classification, including both empirical- and physics-based, as well as hybrid formulations; in Section 3, the mechanisms and modelling of volume change and deformation of different common products/processes are discussed; in Section 4, conclusions and perspectives are given to finalize.

## 2. Modelling Approaches

As it was discussed in the Introduction section, deformation of solid-like food materials is not an isolated phenomenon, but it is definitely coupled with heat and/or mass transport processes, which establish the driving forces for deformation during processing. In other words, in most food engineering applications, deformation of a material does not involve a pure solid mechanics problem, but there is a physics coupling, i.e., it is generally a multiphysics problem. An exception could be the simulation of a texture/TPA or mechanical test, or modelling a “dry” oral breakdown or mastication process. So, in general terms, modelling of deformation is naturally related to modelling of heat and/or mass transfer, or the corresponding transport phenomena for a given process. Nevertheless, since this review is focused on volume change and deformation, only essential discussions regarding modelling of other phenomena during food processing are included, together with relevant references.

Considering that more than one phenomenon or transport process can occur, and thus be modelled and simulated for a given product/process, classification of modelling approaches is not straightforward, since different types of assumptions, simplifications and solutions have been proposed by many authors for several applications. Based on the performed literature analysis, we propose to divide the modelling approaches into two main groups, depending on the complexity involved and the degree of detail provided; within each group, different kinds of modelling approaches are included, from empirical-based to physics-based:

1. Empirical, phenomenological, and simple theoretical models: Overall, these models are relatively simple, in terms of formulation and implementation, and their outputs are average or bulk values. Some models of this group can help in providing local or detailed information, in combination with physics-based models, although using simplifications in the formulation and/or implementation (i.e., hybrid models from the second group).
2. Physics-based and hybrid models: In general terms, these are transport models formulated from physical or fundamental laws, which may involve different and multiple scales. These models provide information about local values (e.g., spatiotemporal profiles), and average values as well. In some cases, complexity is reduced by using some simplifications (hybrid models).

**Figure 1:**
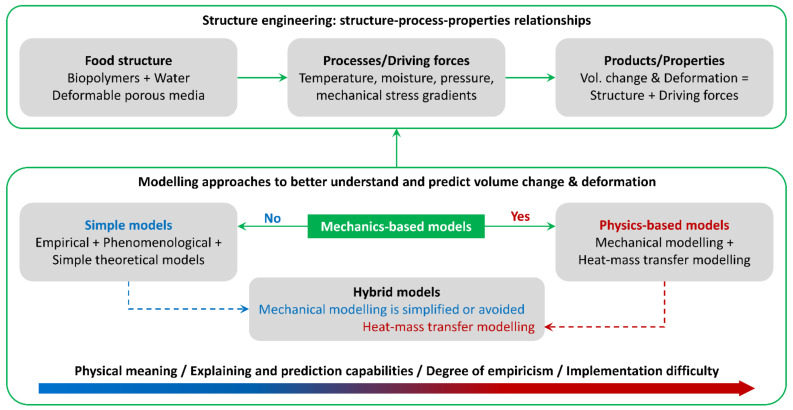
Graphical summary of the review article, including general perspective and modelling approaches, according to the proposed classification.

Following, we present the description and more relevant details of each group of models, prior to discussing a series of application examples in Section 3. Figure 1 depicts a summary of modelling approaches and overall perspective of this review article.

### 2.1. Empirical, Phenomenological, and Simple Theoretical Models

Firstly, empirical modelling approach aims at finding a direct relationship between experimental inputs and outputs (data), without the premise of describing the underlying mechanisms that explain such connection. In this sense, these models are often referred as black-box or data-driven models. Such direct relationship can be established by using different numerical tools, e.g., regression models, response surface methodology, and also more complex methods like artificial neural networks [23]. In our case, inputs would be some product characteristics and processing conditions, while outputs would be different variables or properties associated with food deformation, e.g., ratio of volume change (shrinkage/expansion), density, porosity, etc. The main advantage of this approach is the low–medium difficulty in terms of mathematical modelling, which makes it more easily implementable. This is of particular interest for industrial applications, especially for SMEs (small- and medium-sized enterprises), which may not have access to more sophisticated or complex tools [24]. On the other hand, the major limitation of this approach is probably the lack of a physical meaning of the established relationship, and thus the impossibility of explaining the occurring phenomena. In addition, this approach generally needs a large amount of data for fitting/training and validation, covering a wide range of conditions, in order to provide reliable tools.

Secondly, phenomenological models are based on hypotheses derived from experimental observations about a certain phenomenon or behavior of interest, and attempt to describe the involved mechanisms by relating some key variables and/or parameters. The difference between this approach and the empirical one relies on the degree of fundamental knowledge involved. Pure empirical modelling is basically a data-fitting problem, while phenomenological models can be seen as a first step towards a physics-based or fundamental model, or as a simplified version of it. For instance, strictly speaking, the classical transport phenomena “laws” like Fourier’s law and Fick’s law, among others, are phenomenological relationships. Afterwards, when a phenomenological model is proved to be valid for a wide range of materials/conditions, or it can be derived from theory under certain conditions, it acquires a physical law status, mostly in an engineering context. Phenomenological models are also called as semi-empirical or semi-theoretical models. In general, the empirical aspect of these relationships is lumped into a (effective) coefficient or property, which depends on the material and main variables of the process (e.g., temperature and water content).

Thirdly, it may be possible to derive simple models from fundamental concepts and principles, i.e., from theory. For instance, by taking certain assumptions, mass balances can be used to obtain a simple model either to predict overall volume change or to compute the local velocity of deformation (some examples are covered in the next section). Such assumptions make possible to obtain these so-called simple theoretical models, which can be also helpful to reduce the complexity of physics-based models, as we discuss later.

Overall, the common characteristic of these three types of models is that they are not mechanics-based models. That is, volume change and deformation are not predicted by solving the solid momentum balance equation and using mechanical properties of the material, but certain aspects of these phenomena are described in an indirect or simplified way. In addition, the degree of empiricism can be significant. Nevertheless, it is worth recalling that mathematical models are essentially tools, which may have different objectives. In other words, from a pragmatic viewpoint, these simple models can still be useful for the design, control, and optimization of processes, besides the mentioned use in combination with more complex physics-based formulations. However, the models of this first group have a limited capacity of describing the underlying mechanisms that explain the behavior of the products/processes. Furthermore, empirical-based models are constrained to specific conditions (products/processes) from which empirical parameters were estimated, i.e., new parameters will be needed if new conditions have to be incorporated into the existing model [1]. In this sense, extrapolation or generalization in terms of both behavior or mechanisms and numerical predictions should be avoided without an appropriate experimental verification.

### 2.2. Physics-Based and Hybrid Models

Physics-based or mechanistic modelling approach is based on the use of fundamental physics concepts and laws to describe the physical mechanisms involved in a process. The starting point for physics-based models is the formulation of the problem, i.e., establishing a set of hypotheses regarding the (supposed) underlying physics of a process and developing their mathematical representation via the corresponding physical laws. Mathematical formulation comprises governing equations, typically partial differential equations, with their respective boundary and initial conditions. Governing equations involve balances or conservation laws of mass, energy and momentum, and the corresponding constitutive equations or transport phenomena laws or expressions, according to established hypotheses, e.g., Fick’s law of molecular diffusion, Fourier’s law of heat conduction, Hooke’s law of elasticity, etc. As it was mentioned before, these transport phenomena “laws” are phenomenological, but they have been extensively utilized and are considered of general application. Nevertheless, special attention is needed in food process modelling: The complex structure and composition of foods may generate significant deviations from the ideal behavior of simple or ideal media/materials (e.g., metals and ideal gases). Finally, the model is completed with thermophysical properties, transport coefficients and other parameters. In most cases, material properties are not constant values, but depend on state variables, e.g., water content, temperature, and also porosity in deformable porous materials. For instance, if a physics-based model is oversimplified by neglecting significant mechanisms and/or using effective properties to lump complex behavior, the model is indeed a phenomenological or semi-empirical one, as described above.

Bearing in mind the focus of this review, the objective of a physics-based model is to describe and predict the deformation behavior and associated variables like velocity of solid and spatiotemporal evolution of porosity. However, as it was already mentioned, driving forces for deformation are usually originated by heat and mass transport processes, so physics-based models need to account for all phenomena involved. This generates a coupled system of equations for which numerical implementation is not straightforward (analytical solutions are not possible). Fortunately, due to advance in computing power and the availability of specialized software, solution of these models is feasible nowadays (although not obvious). After model solution, numerical results need to be validated against experimental data, i.e., hypotheses of the model have to be tested properly by using data from the real process. Afterwards, the model hypotheses can be modified to better describe the real behavior and thus to obtain a more accurate model.

To illustrate this approach, let us consider the poromechanics-based modelling framework for the coupled physics of transport and large deformation in food materials, developed by Dhall and Datta [20], based on extended Biot’s theory of poromechanics. In order to provide a concise description, we focus on essential aspects and equations related to large or finite deformation. Firstly, food is considered as a multiphase porous material where all the phases are in the continuum (macroscale approach), and the solid skeleton is an incompressible hyperelastic (or Green elastic) material. This nonlinear constitutive theory is suitable to describe a wide range of physical phenomena in which deformation may be large or finite [25]. Secondly, the macroscopic total stress tensor ($\bar{\sigma }$) can be written as a sum of averages in the individual phase volumes of the material, leading to the following expression:

(1) $$\bar{\sigma }={\bar{\sigma }}^{\prime }-p_{f}\;I$$

where ${\bar{\sigma }}^{\prime }$ is the effective stress on the solid skeleton, and the second term represents the pore or fluids pressure, $p_{f}$ (I is the identity tensor). Thirdly, the solid momentum balance can be simplified by assuming quasi-steady state for deformation (no acceleration) and no external forces being applied:

(2) $$\nabla \cdot \bar{\sigma }=0$$

By combining both equations, we obtain the relationship between effective stress on solid skeleton and driving forces (pressure in the pore):

(3) $$\nabla \cdot {\bar{\sigma }}^{\prime }=\nabla p_{f}$$

For instance, if the pores contain liquid water and gases (e.g., air–water vapor mixture and carbon dioxide produced by yeasts), the governing equations become the following:

(4) $$\nabla \cdot {\bar{\sigma }}^{\prime }=\nabla p_{g}-\nabla \left(S_{w}p_{c}\right)$$

On the right-hand side, the first term is the gas pressure (*p*_g_) gradient and the second term involves capillary pressure (*p*_c_; *S*_w_ is liquid water saturation), which can be related to water potential via Kelvin’s law. Capillary pressure (or water potential) is generally a function of water content and temperature (although temperature dependency is commonly neglected).

In summary, Equation (4) indicates that effective stress on solid skeleton is due to driving forces established by a given heat and mass transfer process. In addition, Equation (4) is related to strains and displacements of solid matrix via constitutive equation of the material (e.g., hyperelastic material). So, Equation (4) establishes that deformation behavior (strains, solid displacements) depends on mechanical properties of the material and driving forces. Considering that mechanical properties can also depend on state variables (e.g., water content and temperature), the multiphysics problem becomes highly coupled. Note that other constitutive equations for the material can be used, but Equation (4) is still valid, since it represents the governing equation for momentum transport. For more details about this general formulation, the reader is referred to Reference [20], and solid mechanics textbooks (e.g., Reference [25]).

In general terms, physics-based modelling approach presents some advantages over the empirical-based modelling [26]:

- Variables, functions, and parameters have physical meaning, so results can be interpreted and explained with certain logic;
- The number of experimental tests is usually reduced, which decreases time and resources involved;
- Great insight into the process is provided due to possibility of performing virtual experimentation and useful studies like sensitivity analysis and “what-if” scenarios;
- Design, prediction, control, and optimization capabilities of products/processes are improved.

Because of these advantages, physics-based models are considered as a key element for the development of digital twins and virtualization of food industry or Industry 4.0 [22]. The drawbacks of this approach are mainly associated with the implementation of physics-based models in food processing. The main bottleneck is probably the lack of data about thermophysical and mechanical properties for a wide range of products and processes [21]. More work is needed in this essential aspect, supported by adequate experimental methods and also by physics-based models, which can be used as estimation tools via inverse methodology. Moreover, the development and implementation of these complex models can be a difficult task, especially in the industrial environment, so more specific or adapted modelling frameworks and software, as well as education and training, are necessary to expand their use in food processing applications [1,24]. For example, advanced numerical methods involving moving meshes are generally required.

So far, previous description is quite general and with focus on the main modelling framework used in food engineering, i.e., continuum or macroscale approach. Briefly, macroscale approach is based on the continuum hypothesis and averaging methods, where a representative elementary volume (REV) is used to describe a “point” of a structureless continuum domain and to define local macroscopic variables, such as macroscopic total stress tensor in Equation (1). More details about this classical modelling framework can be found elsewhere (e.g., References [27,28,29]). Besides, the finite element method (FEM) is probably the most used numerical technique to solve equations of these macroscale models [30]. On the other hand, in the last 10–15 years, novel and promising physics-based modelling paradigms have been applied in the food engineering field: microscale and multiscale approaches. For the sake of simplicity, we use the term “microscale” throughout this work to describe different fine scales, e.g., microscale, mesoscale, and nanoscale. Overall, the microscale approach aims at describing the behavior of microstructures like colloids, cells, polymers, composites, interfaces and molecular arrangements. These complex microstructures are actually the components of the structureless continuum material of the macroscale approach. So, the idea is to model the physicochemical and mechanical changes occurring at these fine scales.

Multiscale models are defined as a hierarchy of interconnected sub-models which describe the material behavior at different spatial scales [28]. This is an interesting approach since biomaterials actually have a hierarchical structure [31]. In this sense, multiscale modelling can provide a solution to the mentioned bottleneck of macroscale modelling regarding physical properties. For instance, a microscale model can be used either to calculate a transport property due to microscopic dynamics to feed a macroscale model or to understand macroscopic responses through variations at microscale. The most important asset of this paradigm is probably the explicit incorporation of microstructure details on the physics-based model. This feature certainly increases the abovementioned possibilities and capabilities of mechanistic models. For example, it may be possible to design a food product with a target texture or mechanical behavior (structure engineering) by using a physics-based multiscale model [8]. This is of great importance for mimicking and/or reformulation purposes in R&D (research and development) applications, e.g., plant-based analogues of dairy and meat products. For more details about this modelling framework, the reader is referred to the excellent review of Ho et al. [28]. An interesting alternative framework for multiscale modelling is provided by hybrid mixture theory (HMT), which has a continuum mechanics basis; this approach has been applied to different food products/processes [32]. With the same objective of incorporating microscale information into macroscale models, application of soft matter approaches can also help to obtain a better understanding and useful insights about the relationships between structure and composition, and properties and macroscopic behavior of foods [33].

In summary, the essential characteristic of physics-based or mechanistic models is that volume change and deformation of the material are described in a direct and explicit manner. This modelling approach is mechanics-based, i.e., the solid momentum balance is used to predict the behavior of the product, based on the mechanical properties of the material and on driving forces established by the process. So, this framework is considered here as the best possible solution towards the global objective of developing scientific knowledge and useful tools to describe and predict the relationships between processing conditions and behavior of food materials.

Despite of the mentioned advantages, formulation and implementation of these models can be a difficult task. Therefore, a common solution to reduce the complexity of these formulations is to avoid the resolution of the mechanical part of the multiphysics problem. That is, deformation of the solid is not calculated through the corresponding momentum balance and mechanical properties, but it is obtained by an alternative way, e.g., by using empirical, phenomenological, and simple theoretical models. In such case, the model is catalogued here as a hybrid model, since the mechanical problem is not solved, but heat and mass transport equations are still considered (Figure 1). In other words, hybrid models are a simplified version of physics-based models. Depending on the degree of simplification and/or empiricism involved, the capabilities of the hybrid model will be reduced in comparison with a full or pure physics-based model.

Finally, it is worth to mention an important aspect of physics-based (and hybrid) models: geometric modelling of food materials at different scales, i.e., the process of creating a digital or virtual representation of the structure/geometry of a real product [34]. After a model is formulated, a virtual domain has to be defined to solve the corresponding equations. For the case of modelling realistic geometries, different imaging techniques are available to perform data acquisition. Ho et al. [28] recommended the use of methods that provide 3D images which can be then converted into 3D solid models, in order to capture all possible information about geometry and structure of materials. In particular, 3D models are able to describe full connectivity of porous materials, which is not possible in 2D models. Such imaging techniques include X-ray computed tomography, optical methods and magnetic resonance imaging. In this regard, Wang et al. [35] indicated that X-ray micro-computed tomography (micro-CT) provides the unique ability to capture intact 3D internal microstructure data without significant preparation of the sample and in a non-destructive way. These authors concluded that geometric models will be significantly improved by using micro-CT data, which will lead to more realistic simulations and more accurate solutions to transport equations.

## 3. Applications in Various Products/Processes

Different driving forces established during processing can cause volume change and deformation of food materials, e.g., removal of water in drying and cooking; water uptake in hydration/soaking; internal pressure or gas-induced expansion in baking, extrusion, and puffing; and mechanical stresses in oral breakdown. The objective of this section is to discuss the mechanisms and the applications of modelling approaches of volume change and deformation in typical food products/processes. The revision of examples is based on the following criteria:

- Food engineering perspective: The goal is to provide an overview about significant contributions, with a focus on physical mechanisms and modelling approaches; details about mathematical formulation and numerical implementation are partially covered.
- More recent and relevant works of the last 10–15 years are included (2005–2021, Scopus database); significant review articles are cited, if available, which may cover older articles.
- Focus on solid and semi-solid (raw) materials.

In this way, we expect to provide an essential review that can be taken as reference or starting point for further research and applications in food product/process engineering. Furthermore, by taking a transversal and comprehensive perspective, we aim at condensing common bottlenecks and challenges shared by different applications, since most of available review articles are focused either on a single product/process or on a specific modelling approach. Finally, since some products/processes have been studied for longer time and by more authors than others, we expect that this study also helps to increase feedback between different applications, in order to improve modelling works in food product/process engineering in a global sense.

The section is organized by unit operations, which in general are associated with a limited range of materials and products. For each case, basic principles of the process and details about structure of corresponding material are given, in order to better understand the mechanisms of deformation during processing. Afterwards, main contributions to modelling are presented, taking as reference the ad hoc classification of models previously discussed. As a reference guide, Table 1 presents a summary of the covered applications, including basic information.

### 3.1. Drying

Drying or dehydration is one of the most common and oldest unit operations in food processing. The main objective is to remove water to a certain moisture content, in order to reduce water activity and thus increase shelf life. Water remotion (or dewatering) can be done by different methods, so there is a wide and still increasing variety of drying techniques and related equipment, from ancient solar and traditional convective hot-air drying, to modern methods like electrohydrodynamic and infrared- and microwave-assisted drying. Overall, fruits and vegetables are the typical food materials subjected to drying for preservation; other examples include grains, mushrooms and meat. We focus then on plant-based materials, due to similarity in structure and thus mechanisms implied in deformation. Fruits and vegetables are mainly composed of water, that is contained in the parenchyma tissue. Parenchyma cells are polyhedral, with thin walls, and they are densely packed together. In this sense, plant-based materials can be thought as pressurized, liquid-filled, closed-cell foams [31]. Briefly, microstructure consists of intracellular and intercellular spaces, and cells walls. Intercellular space is formed by pores and capillaries between cells, that contain a small amount of free water, air and some solutes. Intracellular space refers to the interior of the cells, where the major part of water is located and defined as loosely bound water. Finally, cell walls, made of biopolymers, also contain water (strongly bound water). Drying primarily consists of removal of intracellular water, which can migrate by three pathways: cell to cell, cell to pores, or by cell-wall rupture (to pores) [63,64]. Afterwards, liquid water is evaporated and transferred to surroundings.

Dehydration causes shrinkage and deformation of plant-based materials: Removal of water (usually assisted/accelerated by heating) produces a loss of turgor pressure, thermal- and hygro-stresses, and the collapse of cells, with a consequent loss of shape and structure of tissue. Therefore, shrinkage has a negative effect on the quality of dried products. Besides macroscopic changes in shape and volume, hardness of material is increased, surface cracking may occur and rehydration capability of product can be diminished, mainly due to unbalanced stresses and structural collapse as a result of a defective or non-uniform process [36]. Although significant shrinkage is produced by most drying methods (e.g., freeze-drying and use of vacuum can cause less shrinkage and collapse), it is important to understand the mechanisms leading to the mentioned structural changes in order to better steer the process and to obtain products of better quality. For this aim, mathematical modelling can be very useful; however, the task is not straightforward. Drying presents a “multi-cubed” nature: *Multiphase* transport processes occur at *multiple* scales, where *multiple* physical processes are involved [65]. Next, we discuss different modelling approaches aiming at describe shrinkage and associated changes during drying of plant-based materials. It is worth noting that mushrooms and meat products are also high-moisture, cellular-based, porous, and soft materials, so the general concepts introduced here are also applicable to these food products (e.g., References [66,67,68,69]).

Probably the most common and simplest modelling approach applied in drying involves the empirical correlation between a measure of shrinkage and the average moisture content values of the product. Shrinkage is often expressed by using a relative or reduced dimensional change of volume, area or thickness. A summary of these linear and non-linear empirical correlations can be found in Reference [36]. The following equation is a typical example of this approach:

(5) $$\frac{V}{V_{0}}=b+a\;\frac{X}{X_{0}}$$

where *V* is the volume of sample with average moisture content *X* (dry basis), *a* and *b* are fitting parameters, and subscript 0 indicates initial time. This relationship represents the hypothesis that volume reduction of samples is only due to removal of water, which is known as ideal or linear shrinkage. That is, it is assumed that the material is composed by a deformable or soft solid structure whose pores are filled only by water [70]. In practice, deviations from linear shrinkage have been observed for a wide range of materials and operating conditions. Numerous factors have been indicated as responsible for the non-linear shrinkage behavior: drying conditions, sample shape, structural and mechanical characteristics of material, case hardening, glass transition, and presence and concentration of starch in the food matrix [71]. Some of these factors have been incorporated into the empirical correlations in order to improve fitting results and description of shrinkage phenomenon [36].

Due to (non-ideal) shrinkage and structural modifications, porosity of the material can change during drying, affecting transport processes and quality attributes. In this regard, several models have been developed to predict overall porosity evolution as a function of average moisture content [72,73]. For instance, Khalloufi et al. [74] proposed a phenomenological model considering also the initial air content, besides average moisture content, and two possible phenomena for porosity formation: shrinkage and collapse. Madiouli et al. [75] reported a simple semi-empirical model to calculate the bulk porosity of a material during drying, based on three properties (solid density, liquid density, and initial bulk density) and experimental data of the reduced moisture content (*X*/*X*_0_) vs. the volume shrinkage (*V*/*V*_0_). More recently, Joardder and Karim [76] developed a phenomenological model for porosity prediction by using a heat and mass transfer model for drying and the so-called “shrinkage velocity”, which depends on effective moisture diffusivity and glass transition temperature. On the other hand, it is possible to develop simple theoretical models to relate bulk or average values of moisture content, shrinkage, density and porosity of the material, based on mass balances, density and porosity definitions [77]. In most cases, such models are built upon the assumption of additivity of the volumes of the different phases of the system [36]. Overall, these simple theoretical models are easy implementable and do not require empirical fitting of parameters.

To finalize with the first group of models, it is worth mentioning that artificial intelligence- or machine learning-based modelling has also been applied to predict different aspects of food drying, including prediction of porosity and shrinkage [78,79]. Such models are based on artificial neural networks (ANN) and related algorithms, so they are considered as empirical-based or black-box models. In general terms, an advantage of these ANN methods is the capability of predicting complex non-linear relationships, without using a physics-based model. On the other hand, a large dataset is required for training and validation, and ANN models lack of physical meaning.

Regarding physics-based models, let us first consider the continuum or macroscale approach, which is still the main framework modelling in the food engineering literature. According to the proposed classification in previous section, these models are mechanics-based, i.e., the solid momentum balance and mechanical properties of the material are used to describe deformation during processing. In this regard, the poromechanics-based modelling framework developed by Dhall and Datta [20] is taken as reference work. The authors proposed a comprehensive modelling approach where solid momentum balance is used to relate deformation with driving forces and mechanical properties of the material (e.g., Equation (4)); mechanical, moisture, and thermal strains are considered in the general formulation. Besides, an interesting discussion is given about the importance of the state of the material on modelling shrinkage related phenomena. While the material is in a soft rubbery state, it remains saturated and the gas phase does not enter the pores (water evaporation occurs at surface); this is favored by a non-intensive drying-rate to avoid surface cracking. Then, volume change of food is equal to volume of removed water and free shrinkage assumption can be considered as valid. Under this condition, the solid momentum balance is not required to calculate solid velocity and the multiphysics problem can be simplified by using other methods previously discussed, e.g., mass balances. However, as soft material is dehydrated, the transition to the rigid glassy state occurs, together with shrinkage of pores and increase of bulk modulus. In this case, free shrinkage assumption is no longer valid. Finally, Dhall and Datta [20] highlighted that the main advantage of a solid mechanics analysis is predicting such deviations from ideal or free shrinkage, which allows the prediction of other important aspects, e.g., porosity development, case hardening, and surface cracking.

By using such poromechanics-based modelling framework, Gulati and Datta [37] performed a benchmark study about convective drying of food materials. The physics-based formulation includes the influence of glass transition on mechanical properties of the product (potato). The developed model is able to describe the case hardening phenomenon during drying, as well as to predict various product quality aspects. The authors concluded that deviations from free shrinkage and case hardening are caused by high drying rates, which induce the rubbery/glass state transition and a decrease in the Poisson’s ratio of the material. A similar modelling approach was used by Gulati et al. [80] to understand large deformation during microwave drying. In this case, the model includes Maxwell’s equations for electromagnetics and stresses are caused by pressure gradients. As well as in the two previous cited works, a modified Neo-Hookean constitutive model (hyperelastic material) was chosen to characterize large deformation of food during processing. In addition, all three works utilized the same approach to compute the volume change due to moisture loss: Firstly, according to large deformation (finite strain) analysis [25], a multiplicative decomposition is used to separate the total deformation gradient (F) into a purely mechanical or elastic contribution (F_el_) and a contribution due to moisture effects (F_M_), F = F_el_ F_M_. The elastic deformation gradient depends on mechanical properties and behavior or constitutive model of the material, e.g., Neo-Hookean model. The deformation gradient due to moisture loss depends on the corresponding Jacobian *J*_M_, or volume change due to moisture loss: F_M_ = *J*_M_ I. Secondly, by assuming free or ideal shrinkage, *J*_M_ is calculated as a function of volume fraction of water ($\phi_{w}$), based on a simple mass balance:

(6) $$J_{M}=\frac{V}{V_{0}}=\frac{1-\phi_{w,0}}{1-\phi_{w}}$$

Likewise, porosity can be defined as a function of (total) Jacobian *J* to compute the evolution of material porosity due to deformation [20]. A similar mechanics-based modelling approach was proposed by Aregawi et al. [38] to predict coupled water transport and large deformation of apple tissue during dehydration. In this case, the total strain (ε) is defined as the sum of the mechanical or elastic strain ($\varepsilon_{\mathrm{el}}$) and the shrinkage or moisture strain ($\varepsilon_{M}$):

(7) $$\varepsilon =\varepsilon_{\mathrm{el}}+\varepsilon_{M}$$

The shrinkage strain is expressed as a function of water content (*X*) gradient or difference to a reference state (e.g., initial state *X*_0_):

(8) $$\varepsilon_{M}=\beta \left(X-X_{0}\right)$$

where *β* is the volumetric shrinkage coefficient, defined as follows:

(9) $$\beta =\frac{1}{V}\frac{\partial V}{\partial X}$$

The value of *β* can be obtained from experimental data of *V* vs. *X*; note that Equation (9) is related to Equation (5). Aregawi et al. [38] analyzed different mechanical models for apple tissue deformation behavior during drying: They made a comparison between linear elastic, linear viscoelastic, and nonlinear viscoelastic models. The authors concluded that nonlinear models (Mooney–Rivlin and Yeoh hyperelastic materials) better predict hygro-mechanical behavior, in comparison with linear elastic and viscoelastic models, which are better suited for small deformation (or infinitesimal strain) analysis. It is worth noting that for the case of nonlinear viscoelastic models, the authors also utilized the multiplicative decomposition of deformation gradient, according to large deformation analysis, and the Jacobian due to shrinkage was computed as follows:

(10) $$J_{M}={\left(1+\varepsilon_{M}\right)}^{3}$$

On the other hand, Curcio and Aversa [81] assumed elastoplastic behavior and small deformation for the case of convective drying of potato cylinders. So, the formulation of the mechanical problem was based on Equation (7), and shrinkage strain was defined in a similar manner as in Equation (8). The authors determined experimentally the shrinkage coefficient by considering changes in axial and radial directions, in order to account for anisotropic shrinkage. However, they found similar behavior in both directions, so an average shrinkage coefficient was finally used, corresponding to isotropic shrinkage condition. Besides, mechanical properties depended on local moisture content. Recently, Mahiuddin et al. [82] reported a very useful review about different models used in the literature to describe mechanical behavior of food materials. Mechanical properties and the influence of main aspects of drying on shrinkage were also revised by the authors.

As pointed out by Dhall and Datta [20], a physics-based model can be simplified by avoiding the solution of the mechanical problem; instead, the solid velocity may be calculated by using some of the models of the first group of our classification. These so-called hybrid models are generally used to predict hygrothermal behavior of products, while using a proper formulation that takes into account shrinkage of the material. Since there are many ways of including shrinkage via simple models (e.g., see References [36,70,83]), only a few reference works are mentioned here. For convective drying, Hassini et al. [84] assumed ideal shrinkage and incorporated volume change in a heat-mass transport model via a volumetric hydro-contraction coefficient, which is defined by an expression similar to Equation (5). This volumetric shrinkage coefficient can be related to a linear hydro-contraction coefficient by assuming isotropic volume change. Then, the linear shrinkage coefficient was used to compute hydro-strains in a decoupled mechanical model considering linear elastic behavior. That is, the authors proposed a sequential solution strategy where heat-mass transfer model was not solved simultaneously with the mechanical problem. A similar formulation regarding shrinkage modelling was reported by Hassini et al. [85], also for convective drying, although a viscoelastic model was used and the heat-mass and mechanical models were solved simultaneously. Another hybrid modelling approach was proposed for the case of intermittent microwave–convective drying [86]: The volumetric deformation due to dehydration was calculated by using a phenomenological model for shrinkage velocity, similar to the one proposed in Reference [76]. Then, porosity of the material was related to shrinkage velocity in order to couple deformation with heat and mass transport. A different phenomenological solution to compute solid velocity due to shrinkage during isothermal convective drying was recently applied by Adrover et al. [87,88]: Based on an analogy with swelling of rubbery polymers, a local shrinkage velocity (*v*) was defined as proportional (and opposite in sign) to the diffusive flux of water (*J*_w_):

(11) $$v=-\alpha \left(\phi_{w}\right)\;J_{w}$$

where α is a shrinkage factor that depends on local water volume fraction ($\phi_{w}$). This shrinkage factor can be obtained either from experimental data, by using the same idea of Equation (5), or it can be assumed a priori, e.g., α=1 for ideal shrinkage. This approach was also applied to model continuous and intermittent convective drying of pears under non-isothermal conditions [89]. On the other hand, a CFD–DEM model (computer fluid dynamics for gas flow, and discrete element method for solid phase) was developed to describe fluidized bed drying of grains, where particle shrinkage due to dehydration was incorporated via an empirical equation similar to Equation (5) [90].

To complete this mini-review dedicated to drying, we herein focus on advanced physics-based modelling approaches. For example, Fanta et al. [91] developed a 2D microscale model to predict water transport and large deformation in pear cortex tissue during dehydration under high relative humidity (more than 97%), e.g., water loss during storage of fruits and vegetables. The model considers transport of water in the intercellular space, the cell-wall network and cytoplasm (intracellular space), by using the chemical potential as driving force for water exchange. Regarding deformation, the micromechanics model assumes that turgor loss of the individual cells due to water transport is responsible for shrinkage. The cell wall is modelled as a set of springs and the shrinkage mechanics is described by the Newton’s law. Besides the prediction of microscale dynamics of water transport and mechanical deformation considering a realistic microstructure, the model is able to estimate the apparent water conductivity of the tissue, which can be used in a macroscale model. In this regard, as a continuation of this microscale study, Aregawi et al. [39] developed a multiscale model where the described water transport and mechanical model at microscale was used to estimate apparent properties to feed a macroscale model. In this case, the authors utilized apple tissue as material, subjected also to mild dehydration conditions. At the continuum or macroscopic scale, the mechanics model consists of two parts: nonlinear behavior described by Yeoh strain energy functions, and viscoelastic behavior following Maxwell’s model. A homogenization procedure was used to calculate apparent water diffusion and mechanical properties at macroscale, from simulations with microscale models. As it was mentioned earlier, this is one of the interesting and promising aspects of multiscale modelling approaches, i.e., estimation of macroscale properties from microscale physics-based models. Furthermore, the multiscale approach provides insights about how microstructure of the material affects macroscale behavior. For recent and good reviews about multiscale modelling approach in the context of food drying, the reader is referred to References [63,64,92].

Two more modelling frameworks are worth of mentioning. Firstly, let us consider microscale and multiscale modelling by using meshfree methods, instead of conventional or classical grid-based techniques like finite element method (FEM) and finite difference method (FDM). For instance, Karunasena et al. [93] developed a 2D meshfree particle-based model to predict extreme deformations of cellular structure during drying. In this model, smoothed particle hydrodynamics (SPH) was used to model cell protoplasm as a high viscosity incompressible Newtonian fluid, while discrete element method (DEM) was utilized to model the cell wall as a viscoelastic solid material. The authors also developed a tissue model to describe interactions between cells. Drying was simulated by varying the moisture content, the turgor pressure and cell wall contraction effects, i.e., a moisture content-domain simulation method was proposed, instead of time-domain, due to computational requirements of the method. This microscale meshfree model was then used to analyze the morphological changes of plant-based materials (apple, potato, carrot, and grapes) as a function of cellular properties: cell size, wall thickness, cell wall stiffness, cell wall contractions during drying, turgor pressure, and pectin layer dimensions and stiffness [94]. Recently, the same research group proposed a coarse-grained multiscale model to describe macroscale behavior based on microscale dynamics by using also meshfree methods, based on previous works [95].

Secondly, we briefly introduce a multiscale modelling approach for swelling biopolymers based on the hybrid mixture theory (HMT). This approach considers three spatial scales (micro, meso, and macro), and a continuum thermodynamics-based formulation to describe macroscale behavior based on phenomena occurring at all three scales [96]. At microscale (microns), the solid biopolymers and vicinal fluid (solvent, e.g., water) exist as separate phases; at mesoscale (millimeters), solid biopolymers and vicinal fluid form a homogenous mixture, and coexist as a separate phase with two bulk fluids (e.g., water and oil); at macroscale (centimeters), a homogeneous mixture of different phases is considered. The main advantage of this approach is the possibility of predicting non-Fickian/non-Darcian fluid transport in the vicinity of glass transition. Since rubbery/glassy state transition is common in food materials during drying, this theory from polymer science appears as interesting and well-suited. For example, this approach was applied to predict water transport and stress development in corn kernels during drying, assuming viscoelastic behavior for the material [97,98]. Recently, the same multiscale framework was proposed to model moisture transport in strawberries and carrots during drying; the HMT-based fluid transport equation was coupled with product quality and nutritional attributes for a comprehensive description of the effects of drying on overall product quality [99]. It is worth to note that uniform (no variation in shape) and also ideal shrinkage was generally assumed in these works, together with viscoelastic behavior.

So far, it is evident the wide spectrum of modelling approaches that have been applied to predict volume change and deformation of food materials during drying. In this sense, this traditional and (still) important process can be considered as a benchmark problem in food engineering, and it may help us to follow the evolution of modelling approaches. We have attempted to provide a comprehensive overview in this regard: from simple empirical and theoretical models aiming at predicting overall shrinkage to physics-based models, which, in turn, have also evolved from classical continuum or macroscale framework to microscale and multiscale approaches, also involving modern meshfree methods like SPH and DEM. Furthermore, interesting and well-suited concepts and techniques from other fields, e.g., soft matter, polymer science, and particle technology, have been used to develop more accurate models, capable of explaining complex phenomena at different spatial scales. In consequence, and for sake of simplicity, we take this subsection as a reference for the following applications.

As a partial conclusion, we understand that there are still some bottlenecks to deal with, especially regarding physics-based models. As we have already mentioned, an important problem to tackle is the availability of transport and mechanical properties of materials for an appropriate range of operating conditions, including temperature variation. In this sense, an interesting research was recently published by Khan et al. [100]: Nanoindentation experiments were performed to study the relationships between mechanical properties and moisture content of plant-based materials during drying. Another bottleneck is experimental validation of simulation results. In this case, X-ray micro-computed tomography appears as a very powerful tool, which can be used also to estimate structure related properties. For example, Prawiranto et al. [101] utilized this imaging technique to characterize and quantify the changes of the microstructure of apple tissue during drying under natural convective, forced convective and coupled irradiation–convective drying. More work in this direction will certainly help to improve physics-based models, in order to obtain a more accurate prediction of mechanical behavior of materials during drying.

### 3.2. Hydration/Soaking

Contrary to drying, hydration is the process of increasing the water content of a material. This operation is an essential step in several processes involving grains (cereals and legumes), which are generally harvested dry. Soaking generates positive effects on the physicochemical and nutritional aspects of grains, and it is required for subsequent industrial operations, such as cooking, extraction, fermentation, germination and malting. For instance, hydration helps to reduce the cooking time of grains (e.g., beans and rice), and facilitates the homogeneous gelatinization of starch and denaturation of proteins during cooking, besides improving the inactivation of anti-nutritional factors [40]. From the transport phenomena perspective, hydration is a mass transfer process driven by difference in water activity and depends on structure and state of the material. In general terms, grains present a complex and heterogeneous structure with different tissues and components, so diffusion may not be the only water transport mechanism, e.g., capillary flow through pores and channels plays an important role during hydration [40]. Furthermore, considering starch-rich materials, diffusion can be classified into three categories, depending on the value of *n* in the relation $x_{w}\propto t^{n}$, where $x_{w}$ is the fraction of water taken by solid matrix and *t* is the diffusion time [41]: (i) *n* = 0.5, Fickian diffusion in rubbery state; (ii) *n* ≥ 1, diffusion in glassy state; and (iii) 0.5 < *n* < 1, non-Fickian diffusion near glass transition.

Water absorption results in a significant expansion of the material, i.e., swelling [42]. This phenomenon is produced at microscale due to incorporation of water into the grain microstructure formed by biopolymers like proteins and starch, and it is macroscopically observed by changes in volume/shape of grains, together with variations in texture (softening). At the same time, swelling of biopolymers can affect water transport due to changes in mechanical behavior. In this regard, when hydration is carried out at high temperatures (>50–60 °C), starch gelatinization and protein denaturation may occur, increasing the complexity of the process [41]. Due to its industrial relevance, it is important to model the hydration of grains, in order to better design, optimize and control the process. In this sense, different approaches have been applied, considering the swelling phenomenon, and are discussed next.

Firstly, let us consider empirical models aiming at predicting the evolution of overall swelling of grains during hydration processes. For instance, empirical-based relationships commonly used to predict water uptake during soaking have been applied to follow dimensional changes of grains. Yadav and Jindal [102] tested two relationships for modelling the expansion of rice kernels during excess water cooking as a function of time, e.g., exponential equation and Peleg’s equation, but finally proposed a power-type model to predict relative expansion due to amount of water uptake. The authors found that swelling was not uniform, i.e., more expansion was registered in lateral direction in comparison with longitudinal direction, and reported that higher expansion occurred in high amylose rice varieties. That is, swelling was not isotropic and depended on structure aspects of rice kernels. Likewise, Hu et al. [103] evaluated five models to fit expansion ratio of rice grains as a function of soaking time, at different temperatures (25–70 °C): Peleg’s equation, solution of diffusion equation (exponential function), Weibull model, and two different sigmoidal equations. Overall, good fitting results were obtained in all cases, but models lack of physical meaning and parameters depend on specific experimental conditions.

On the other hand, Sayar et al. [43] utilized two approaches to model the linear (length, width, and thickness) and volumetric expansion of chickpea seeds as a function of water uptake during soaking at different temperatures (20–100 °C). The first approach was based on the experimental correlation between volume variation and water absorption of chickpeas during soaking, expressed as follows:

(12) $$V-V_{0}=\lambda \frac{\left(M-M_{0}\right)}{\rho_{w}}$$

where *V* is the volume of chickpea at time *t*, and *V*_0_ its initial volume; *M* is the weight of chickpea at time *t*, while *M*_0_ its initial weight; *ρ*_w_ is the density of water, and *λ* is the volumetric expansion coefficient. If *λ* = 1, the volume increase is equal to volume of absorbed water, i.e., ideal swelling or volume additivity assumption (similar to ideal or free shrinkage previously discussed). However, all values found were smaller than 1, e.g., 0.73–0.95 for different temperatures. The second approach involved solving Equation (9), previously introduced to define the volumetric shrinkage coefficient. In this case, different values of the expansion coefficient (*β*) were obtained by using volume, length, width and thickness variation of chickpeas, indicating anisotropic swelling. Furthermore, Sayar et al. [43] analyzed the variation of different expansion coefficients with temperature: Overall, all coefficients decreased linearly in the range of 20–50 °C, and then remained constant for 70–100 °C. The authors indicated that starch gelatinization occurring at around 60 °C would explain this behavior in swelling of chickpeas.

Secondly, we summarize physics-based and hybrid models applied to predicting swelling of grains. Considering macroscale or continuum framework, some researchers have applied a formulation similar to the one described by Aregawi et al. [38] for modelling coupled water transport and small deformation during dehydration, i.e., Equations (7)–(9). For instance, Perez et al. [104] utilized a realistic 3D geometry of rice obtained from tomographic images to simulate hygroscopic swelling during soaking at different temperatures. Hooke’s law (linear elasticity) was used to model elastic strain of material, and Fick’s law to describe water transport. The authors aimed at better understanding the development of internal stresses due to swelling that leads to cracking and breakage of grains during soaking [44]. Through the proposed model, it would be possible to optimize the soaking process in order avoid breakage, which may result in loss of texture and thus of quality of rice. A similar formulation was also used to model water uptake of yellow peas during the steeping (soaking) step of a malting process, with the objective of selecting optimal time–temperature conditions of this critical stage [105]. In this case, it was assumed that the pea remains spherical during hydration (uniform expansion) and behaves as an elastic material, i.e., Hooke’s law was utilized for the stress–strain relationship. Another example involving the mentioned formulation was reported to model water uptake and hygroscopic swelling of dehulled barley grains during cooking of canned porridge [106]. Again, linear elastic behavior was assumed, but mechanical properties were considered as functions of glass transition temperature. As we mentioned above, these models did not utilize a large deformation framework, but considered small deformation of grains.

Other researchers have applied more complex concepts and approaches to model and better understand the swelling of materials. For instance, a two-scale thermomechanical theory for unsaturated swellable porous material was developed by considering large deformation and viscoelastic behavior of the solid matrix [107]. Then, this theory was applied to model boiling of pasta, i.e., soaking at boiling temperature [108]. Another interesting approach was developed by van der Sman [109]: A novel Lattice–Boltzmann method with a deforming lattice was used to model one-dimensional swelling of gel-like materials; the model assumed that volume changes are only due to loss/gain of water. The author aimed at providing an adequate description of the swelling of cell wall material for a further development of a multiscale simulation framework for hydration of porous foods. Besides, a good introduction to the Lattice–Boltzmann method is given in Reference [109]. The last example of these complex physics-based approaches involves the use of a soft condensed matter perspective to model hydration kinetics of navy beans [110]. The Flory–Huggins equation was employed to describe the osmotic pressure produced by the polymer–solvent mixture (i.e., protein–water), and the swelling was modelled as a moving boundary problem by assuming volume additivity. It is worth noting that the last two examples can be considered as hybrid models in this work, since volume change was not described by using a mechanical model. Nevertheless, we think that these advanced concepts and frameworks, which are mainly applied in other fields, can be an inspiration to developed physics-based models in food engineering.

Finally, let us consider macroscale hybrid models for swelling of grains. Overall, the following examples have assumed water transport by Fick’s law, using an effective moisture diffusivity, and volume change was modelled in a simplified way, without a mechanical formulation. For simplicity, we focus on how the authors proposed to solve the modelling of volume change. For the case of excess water boiling of rice, and considering an ellipse as geometry, an empirical-based linear relationship between dimensions and moisture content of grain was proposed by Bakalis et al. [111]. This relationship was used to update the simulation domain at each time step, according to water uptake. A similar solution was used by Nicolin et al. [112,113], although the empirical relationship involved radius of sphere and time of hydration. In this sense, Pramiu et al. [114] proposed a physically consistent simple expression for variation of average grain diameter with soaking time, considering values at initial and equilibrium times of hydration. On the other hand, uniform swelling and volume additivity were assumed to generate an equation for the variation of sphere radius with time to model soaking of rice [42,115]. Similar assumptions were established by Briffaz et al. [116] to relate Eulerian and Lagrangian frames to calculate solid velocity due to swelling. Finally, Nicolin et al. [117] also used a mass balance to derive a differential equation for radius variation with time, but they included the expression of diffusive flux evaluated at surface to account for all mass accumulation inside the grain.

In summary, we found that there are some gaps to fill in the modelling of hydration/soaking of grains, especially considering physics-based models at macroscale. More research is needed considering the following aspects: large deformation analysis and nonlinear mechanical models; non-ideal and anisotropic or non-uniform swelling; and influence of glass transition and composition on mechanical properties of grains. It is worth recalling the importance of developing accurate models to better design and optimize this process, since it is applied to staple foods like rice and plant-based protein-rich products such as legumes.

### 3.3. Cooking/Roasting

Cooking is a general term referring to the transformation of a raw material into a ready-to-eat food, mainly by application of heat. Any cooked product needs to be microbiologically safe and acceptable regarding sensory features, e.g., texture, color, and flavor. Although several foods are subjected to cooking in a general sense, we focus here on the cooking/roasting of meat products, since other food materials are covered in other subsections, e.g., vegetables in drying (Section 3.1.), grains in hydration (Section 3.2.), bakery products in baking (Section 3.4.), and snacks in extrusion and puffing (Section 3.5.).

In particular, cooking of meat is essential to obtain a safe and appealing product. Meat products are approximately composed of 20% of proteins that represent the main constituent making up the structure of a meat product. During the cooking process, the proteins undergo substantial structural changes affecting the quality of the final meat product [118]. Particularly, meat proteins denature and cause structural changes, such as the shrinkage of muscle fibers and connective tissue [119]. Changes in muscle fibers during cooking in the 45–90 °C range occur in two phases: At about 45–60 °C, the shrinkage is primarily transversal to the fiber axis, and at 60–90 °C, mainly parallel. At a higher temperature of about 121 °C, there may be a third shrinkage of meat which is transversal to the fiber axis [118]. The structural changes affect the water holding capacity of the meat: The mechanical force exerted by the contracting protein network on the interstitial fluid, denoted swelling pressure, leads to the expulsion of the water from the meat [9]. Darcy’s law was used to associate the hydraulic pressure with the moisture transport [120]. As the temperature increases during cooking, a pressure gradient builds up and induces fluid motion, deformation, and, consequently, shrinkage of the solid matrix. The shrinkage of meat is one of the most important physical changes occurring during the cooking processes [121]. Besides the mentioned relationship between structural modifications of proteins during cooking and quality of final products, shrinkage is also important for calculation of cooking times, due to changes in volume and shape affecting the computation of concentration and temperature gradients. Overall, it is important to understand the mechanisms underlying deformation during cooking and their relationship with other phenomena. Next, we provide an overview of mathematical approaches proposed to deal with this relevant problem in food engineering.

The shrinkage during meat cooking can be taken into account by considering that the change of dimensions is proportional to the moisture content [66,70,121,122], or by considering shrinkage as the integrated result of temperature-dependent and volumetrically distributed shrinking [123]. For instance, Clemente et al. [66] determined shrinkage evolution for pork meat during drying and reported a good linear relationship between the *V*/*V*_0_ ratio and the moisture content. This relationship was found to be independent on the size of the samples, their salt content, or drying conditions. In general terms, the water losses are reported as the main responsible for shrinkage. Wang et al. [122] evaluated the shrinkage of chicken nuggets during deep-fat frying: Linear fitting of volumetric shrinkage vs. moisture loss gave values of the coefficient of determination (*R*^2^) between 0.90 and 0.94. Du and Sun [121] investigated possible correlations between shrinkage and water content of pork ham by using computer vison data. They found that the total volume shrinkage was highly and negatively correlated with water content (*r* = 0.98). It is worth noting the similarity between these empirical approaches to model shrinkage of meat during cooking with the ones discussed for volume changes during drying and hydration.

Concerning the physics-based modelling of meat cooking process, the underlying physical phenomena involve the coupling of heat and moisture transfer in a deforming porous medium [3]. A quite limited number of models were developed about meat cooking/roasting and two different approaches were investigated to describe mass transfer inside meat; the first one considering only diffusion [124,125,126,127], and the second one describing the moisture transport by the Flory–Rehner theory [45,119,120,128,129]. However, little information has been provided on modelling meat deformation during cooking. Considering mechanics-based models, we should mention again the poromechanics-based modelling framework developed by Dhall and Datta [20]: Contact heating of a hamburger patty was taken as an example of application of the general modelling approach. Briefly, large deformation analysis was performed (multiplicative decomposition of deformation gradient), meat was assumed to behave as an hyperelastic material (Neo-Hookean model), and free shrinkage was considered to calculate the Jacobian due to moisture loss, e.g., Equation (6). This approach was recently used by Moya et al. [46] to develop and validate a numerical model able to simulate the double-sided pan cooking of beef. The proposed model takes into account the heat flow from the pan to the meat and the moisture transfer simultaneously with the meat deformation. The model considers the swelling pressure gradient caused by the shrinkage of the meat fibers and connective tissue, due to the denaturation of proteins and the loss of the water holding capacity during cooking.

In addition, some authors have proposed hybrid models, i.e., shrinkage was solved in a simplified way. For instance, Zorrilla and Singh [130] developed a mathematical model to predict temperature profiles in meat patties during double-sided cooking, assuming a 2D cylindrical geometry where the radial shrinkage changed with temperature. To account for shrinkage, two reductions in the patty diameter were evaluated, e.g., 13% and 18%. Considering the oven roasting of meat, Feyissa et al. [47] proposed a 2D mathematical model of coupled heat and mass transfer. Regarding shrinkage, the authors formulated an expression based on a simple mass balance to relate the volume of water removed (*V*_w_) with shrinkage of meat, represented by volume, *V*:

(13) $$V=V_{0}-\beta \;V_{w}$$

where *β* was used to describe the effect of the formation of pores during roasting, and it can vary between 0 (the volume of water lost is entirely replaced by air and no deformation occurs) and 1 (the volume of water removed is equal to the volume deformation, i.e., ideal shrinkage). Finally, Blikra et al. [48] studied the shrinkage of cod filets and loins during oven heating at high relative humidity. Shrinkage was modelled by using a semi-empirical approach: Volume reduction was assumed to be due to cook loss, i.e., liquid exudate dripping from the fish during heating, which was obtained empirically.

So far, we can say that drying of fruits and vegetables, hydration of grains, and cooking of meat products have been treated similarly regarding modelling of volume change and deformation, considering all modelling approaches: Overall, shrinkage or swelling is assumed to be due to water loss or gain, respectively. This behavior has been supported by different experimental studies, including materials and process conditions. The reason for this conclusion relies on the structure of materials: Cellular solids made of biopolymers are filled (or to be filled) with a large amount of water. In addition, hygrothermal changes generate important transitions that affect mechanical behavior and heat-mass transport: Glass transition, starch gelatinization, and protein denaturation. These remarks can be considered as positive towards a common modelling framework and transversal solutions. However, as we mentioned before, more work is needed regarding specific mechanical properties of materials under real process conditions.

### 3.4. Baking

Baking is the final and most important step in the production of bakery products such as breads, cakes and biscuits. During the baking process, simultaneous and coupled physical, chemical, and biochemical changes occur in the products, which are responsible for their final overall quality [131]. Inside the oven, heat and mass transport generates variations in temperature and moisture content of a product, that are responsible for physicochemical and biological transformations such as browning reactions, evaporation of water, crust formation, volume expansion, gelatinization of starch and denaturation of proteins, which make baking a complex process [132,133,134]. A rapid increase in overall volume at the beginning of baking (so-called oven rise) was reported in several experimental studies [135]. Increase in gas pressure is the driving force to explain expansion [136]. Bakery dough initially includes unconnected gas bubbles mainly filled with carbon dioxide generated by yeast (or chemical leavening agents). When the bubbles grow with the release of CO_2_ and the temperature increases, they come into contact and gas transfer becomes possible. Bread swelling induces an increase of porosity. Rheological properties have a significant effect on the deformation; gelatinization happens at about 60 °C and the dough turns into crumb. With the appearance of the dehydrated crust at surface, the deformation is constrained due to the outer solid/rigid structure, especially in traditional bread making (e.g., French bread).

Considering the importance of mechanical/rheological properties for the deformation phenomena in bakery products, Guessasma et al. [8] elaborated a review about the mechanical modelling of cereal solid foods. The authors stated that it is possible to predict material properties from the accurate knowledge of its structure. Mechanical behavior of a solid cereal food is mainly affected by the water content (water is the plasticizer that governs the glass transition and also starch gelatinization, together with temperature) and by the structural characteristics (micro-structural and meso-structural levels). Besides water content and structure, density and porosity are the main parameters that explain the variations of the mechanical properties of cereal foods [8]. Therefore, baking appears as a multiphysics problem where simultaneous and coupled heat and mass transfer produces the expansion (large deformation) of the porous structure of dough, which is driven by pressure gradient. In addition, state transitions of biopolymers (starch and proteins) are part of this complex process, which determine also the final structure and texture of the products.

In order to better understand the underlying mechanisms of baking, and thus improve its design and the overall quality of bakery products, different modelling approaches have been applied. In general terms, the baking models can be classified into two categories: diffusive or phenomenological models [49,137,138,139,140,141,142,143,144,145], and multiphase or physics-based models [50,51,52,53,146,147,148,149,150,151,152,153,154]. In the first case, only temperature and moisture content are calculated, i.e., liquid-water and vapor-water phases are not separated, and production of CO_2_ is not taken into account. Therefore, these models cannot predict variation of pressure inside the product, and thus cannot describe expansion of porous matrix by a mechanical or physics-based approach. Instead, volume expansion can be included by using empirical correlations obtained from baking experiments. For instance, Purlis and Salvadori [49] utilized a moving mesh method where velocity of deformation at boundary was described by experimental volume change of bread during baking. A similar approach was applied for modelling baking of sponge cake [145].

On the other hand, physics-based models take into account the mass conservation for each phase and the gas pressure can be introduced and predicted [53,152]. Consequently, deformation can be expressed as a function of the gas pressure change, e.g., by using the solid momentum balance with pressure gradient as driving force, as shown previously in Equation (4). In addition, evolution of porosity can be predicted, in order to describe structure variation during baking. For instance, Zhang et al. [154] and Zhang and Datta [53] first developed a multiphase heat and mass transport model for bread baking, where large deformation was considered by using the principle of virtual work. Driving force for deformation was assumed to be internal pressure, and bread was modelled as a viscoelastic material (Maxwell’s model). In addition, the relaxation time (rheological property of the viscoelastic model) was expressed as a function of temperature, in order to represent the dough/crumb transition due to starch gelatinization at 65 °C. Afterwards, Nicolas et al. [151] proposed a model taking into account the heat and mass transfer and the phenomenon of swelling during traditional baking of French baguette bread. The model included the conservation equations of energy and mass to evaluate the water content, pressure, porosity and temperature of bread. A momentum conservation equation was used to calculate the swelling velocity of the porous matrix, which was also considered in the calculation of heat and mass fluxes (i.e., contribution due to solid movement). Particularly, the authors considered the bread like a Newtonian fluid, applying a viscous model. Later, this research group proposed a similar model of bread baking, but a viscoelastic model with a Terzaghi effective stress was used to describe the swelling velocity [52,152]. Variable elastic modulus and time relaxation as a function of the product state (dough, crumb, and crust) were employed. Considering the gas pressure as driving force of the expansion, a mechanical equilibrium between the product and gas pressure was imposed. It is worth noting that these models can be considered as elaborated or adapted applications of the general poromechanics-based modelling framework developed by Dhall and Datta [20], which was previously discussed. Recently, a multiphase flow modelling approach was used to describe the bread baking process in an industrial convection oven [146]. This CFD model utilized a simplified approach to describe volume expansion: Only the middle part of bread was allowed to be deformed (vertically upward), via the (fluids) volume additivity assumption, and the rheological behavior of solid matrix was not considered.

Finally, a few authors have attempted to model microscale phenomena during baking, e.g., transport at pore or bubble level. Bikard et al. [147] proposed in a first step a model to predict the development of porous structure of dough during proofing. For this aim, the authors considered an elementary volume of dough (ca. 1 mm^3^), composed by two phases: solid matrix and gas resulting from fermentation of yeast. For simulation, it was assumed a random distribution of initial bubbles created during mixing/kneading before the proofing step. Then, the model consisted in conservation equations for mass and momentum for each phase, i.e., matrix and *N* number of bubbles. Evolution of 3D foaming was obtained via simulation of the proofing stage. In a subsequent work, Bikard et al. [50] aimed to simulate the baking process by taking the final dough structure obtained after proofing as starting point. In addition to previous model, the authors incorporated the heat balance equation and the thermosetting kinetics of the dough (dough/crumb transition). Likewise, Narsimhan [150] included the diffusion of CO_2_ generated by fermentation, and coupled bubble expansion dynamics to heat and mass transport. Lucas et al. [149] developed a new multiscale formulation that accounts for evaporation–condensation–diffusion of water while pores are closed, and for Darcy flow when pores open. Pores or bubbles opening was assumed to depend on temperature (around 50 °C). At the macroscale, coupled deformation and multiphase heat and mass transport were considered.

So far, it can be said that modelling of expansion during baking may present another degree of complexity in comparison with moisture-induced shrinkage and swelling. In drying, hydration, and cooking, it is possible to simplify a mechanical model without incorporating empirical-based shortcuts, by assuming ideal or free shrinkage or swelling, which actually has been experimentally verified for a wide range of operating conditions and materials. In the case of pressure-induced expansion during baking, prediction of pressure (exerted by different gases, including CO_2_ from fermentation) is obviously required, as well as solving the solid mechanics equation. In addition, different transitions and changes related to intrinsic structure of dough have to considered, e.g., stiffening of the matrix due to dough/crumb transformation, development of the crust (which can be thought as another material or transition), evolution of porous structure (pore expansion, coalescence, and opening), and profiles of porosity (especially near and in the crust). In summary, modelling volume change and deformation during baking is still a great challenge, particularly regarding microscale phenomena towards an accurate description of cellular structure evolution during the process.

### 3.5. Extrusion and Puffing

A wide variety of ready-to-eat foods such as snacks and breakfast cereals are produced by expansion of starch-based matrices. Expanded products can be obtained either by extrusion cooking (direct expansion) or by puffing operations, such as frying or microwave heating (indirect expansion). In all cases, the final products are characterized by a low-density, cellular–porous glassy structure (solid foam), that provides specific texture properties, e.g., crispness. On the one hand, extrusion cooking, or simply extrusion, is carried out in an extruder, composed by three main elements: barrel, screw(s), and die. Briefly, a powdery material or flour is introduced in the barrel, together with water, which is then subjected to mechanical stresses and heat flow to cause the melting of the material, which is transported as a highly viscous fluid towards the die exit. At the end of the extruder, pressure in the fluid is very high (ca. 4–8 MPa); so, after passing through the die, pressure drops and expansion occurs due to instantaneous vaporization of water [155]. Expansion at die exit is a complex phenomenon involving phase transitions and multiphysics at micro- and macroscales, in a very short time interval (less than 1 s): Bubble nucleation and growth, coalescence, shrinkage, and final setting when the molten matrix becomes glassy after cooling [156]. Water plays an essential role in the expansion mechanism by extrusion, acting as a plasticizer for melting (glassy/rubbery transition) and as a blowing agent for expansion [157].

On the other hand, puffing can be defined as the expansion of a pre-structured material (pellet) or a grain (corn and rice) by application of heating, e.g., microwave and frying. Similar to extrudate foods, the final structure of puffed products depends on the glassy/rubbery transition of the material, expansion due to water vaporization, and final rubbery/glassy transition for structure setting [158]. For instance, expansion of starchy pellets during microwave heating involves the following steps: Drying/popping, nucleation, expansion, cell opening, rupture, shrinkage, fixation, and end of heating [54].

Due to its industrial relevance, driven by an increasing demand of healthier snacks and breakfast cereals by consumers, expansion by extrusion and puffing has been subject of numerous studies. The main objective is to better understand the underlying mechanisms of these processes, i.e., the relationships between formulation, processing conditions, and final structure and properties of the products. Next, we provide an overview about modelling efforts regarding this aim. Please note that given the complexity of the expansion phenomenon and space limitation of this article, only brief discussions are included. For a further and more detailed study of this topic, the reader is referred to the following excellent works, References [54,55,156,157,159,160].

Let us first consider empirical-based and phenomenological modelling approaches. For instance, Cheng and Friis [161] utilized classical dimensional analysis (Buckingham’s pi method) to develop a phenomenological model to correlate operating conditions of a twin-screw extruder with product expansion. Similarly, response surface regression together with genetic algorithms were proposed to develop a design tool, so screw speed and temperature can be related to different final product characteristics such as expansion ratio [162]. For microwave puffing of rice, Dash and Das [163] developed a genetic algorithm based on ANN modelling to investigate the effect of microwave power, puffing time, and addition of butter and sodium bicarbonate, on the expansion ratio and puffing percentage of products. With focus on cereal-based extruded foods, Kristiawan et al. [55] carried out a comprehensive study to improve the understanding about the effect of extrusion variables and material properties on the vapor-induced expansion phenomenon. After a detailed analysis, the authors built a helpful conceptual map to describe the relationships between input variables at the die (product temperature, moisture content, melt rheological behavior, and die geometry), and output variables regarding the product (foam density and anisotropy factor). Based on this work, Kristiawan et al. [56] proposed and validated a phenomenological model of expansion to predict the volumetric and radial expansion indices, and the anisotropy factor of extruded products (expanded maize starches), from rheological properties of the melt and thermomechanical conditions of the extrusion process. The model can be used for optimization purposes, or it can be coupled with a 1D extrusion model to build a global model for the design of cereal-based extruded foods [55].

Regarding physics-based modelling at macroscale, mainly puffing has been studied. For instance, Rakesh and Datta [164] aimed at describing puffing during microwave heating. The authors followed the previously discussed poromechanics-based modelling framework proposed by Dhall and Datta [20]: In this case, large deformation was driven by excessive internal pressure due to water vaporization. Likewise, mechanical behavior of the material (potato) was described by a hyperelastic Neo-Hookean model. However, constant mechanical properties were used, and the glass transition was not taken into account to model expansion. These aspects were improved in the subsequent work of Gulati and Datta [57], where salt-assisted puffing by toasting of parboiled rice was studied. In this improved model, it was assumed that solid skeleton undergoes large elastic and inelastic deformations, so it was modelled as a hyperplastic-perfectly plastic solid, i.e., deformation gradient (F) is composed by an elastic component (F_el_, with hyperelastic behavior) and by a plastic component (F_pl_): F = F_el_ F_pl_. A two-parameter Mooney–Rivlin material model was utilized to describe such behavior. In addition, elastic modulus and Poisson’s ratio were expressed as functions of glass transition temperature to account for rubbery/glassy transition.

On the other hand, important and interesting efforts have been made to describe the expansion phenomenon at microscale and to further develop multiscale models. Overall, multiscale models for vapor-induced expansion are based on the coupling of a “cell model” that describes bubble growth dynamics at microscale, with a continuum approach model for transport phenomena at macroscale. Eventually, this multiscale model can be coupled with a model describing the flow behavior inside the extruder. For instance, the cell model developed by Schwartzberg et al. [165] has been used in various subsequent multiscale formulations. Examples of this multiscale modelling approach include the works developed by Manepalli et al. [58], van der Sman and Broeze [166], and Wang et al. [167]. Likewise, Ditudompo and Takhar [168] utilized a two-scale multiphase model based on the hybrid mixture theory (HMT, previously described), coupled with poroviscoelasticity equations to describe transport processes and mechanical changes in extruded products during expansion.

In summary, the vapor-induced expansion during extrusion and puffing is a very complex phenomenon, and its modelling represents a challenging problem for food engineers. In fact, modelling approaches seem to be divided into two main groups: empirical-based and phenomenological models, and advanced physics-based models; the so-called hybrid models are lacking, as well as simple models to predict overall expansion (similar to the case of baking). That is, either the problem is solved in a pragmatic way or complex models are required. Furthermore, experimental validation of physics-based models is not an easy task, since expansion phenomenon and structuring of products occur in a very short time interval and under conditions which are difficult to monitor. In this regard, X-ray microtomography (XMT) imaging has demonstrated to be extremely helpful to characterize and quantify the 3D cellular structure of extruded and puffed materials [169,170,171].

### 3.6. Oral Processing

The ultimate transformation of a food product is its consumption, i.e., human processing. In brief, consumption or eating involves two subsequent major steps: Oral processing (oral digestion) and (gastric) digestion. Oral processing aims at producing the bolus after a series of mechanical and enzymatic processes. Afterwards, the bolus is swallowed and breakdown and absorption of food components occur at gastric level [172]. Oral processing is the most important step for perception and appreciation of texture of foods; both physiology aspects and intrinsic properties of food materials play a relevant role in this complex process [59]. That is, texture is a fuzzy concept since it is a sensory perception derived from the structure of food at different levels and interaction with digestive and cognitive systems. Besides sensory aspects, food texture also plays an important role in controlling food consumption (satiation) [60]. Therefore, in order to design more nutritious, healthy, and enjoyable foods, it is essential to better understand the relationships between food structure, patterns of oral processing, and sensory texture perception. Furthermore, such understanding is also important to improve the design and optimization of processes, since food structure is indeed built up by different processing steps (structuring or structure engineering), as discussed in previous applications. In this regard, modelling approaches and, in particular, mechanical- or physics-based modelling, can help in gaining insights on the structure–properties–oral processing relationships.

In this last application of products/processes, we aim at giving a big picture about modelling of oral processing and its relation with food structure and texture. It is worth to mention that oral breakdown of solid foods involves large deformation but also fracture dynamics, certainly increasing the complexity of (mechanical) modelling, in comparison with previous applications. Considering empirical-based approaches, probably the most popular methodology is texture profile analysis (TPA). TPA aims at reproducing the chewing or indentation by using different settings; response to applied deformation is reported by parameters associated with texture, such as hardness, firmness, crunchiness, cohesiveness, etc. Afterwards, these texture indices can be used to develop kinetic models and correlations to include aspects of processing [12]. TPA is extensively applied in the food engineering field, but it is not very helpful to understand the structure–properties relationship due to the lack of well-defined physical/mechanical parameters [61]. Regarding semi-empirical approaches, scaling law or Gibson–Ashby model is widely utilized to characterize cellular solids [11]. This approach allows predicting mechanical properties (e.g., Young’s modulus, *E*) based on the relative density, defined as the ratio between the density of the foam or cellular solid (*ρ*) and the density of the solid phase (*ρ*_s_):

(14) $$\frac{\mathrm{Foam}\;\mathrm{property}}{\mathrm{Solid}\;\mathrm{phase}\;\mathrm{property}}=C{\left(\frac{\rho }{\rho_{s}}\right)}^{n}$$

where *C* and *n* are empirical parameters to be fitted, which in turn can be related to structure of the porous material. For example, this approach has been used to study the influence of cellular structure, given by formulation and dough processing conditions, on the mechanical properties of bread crumb [173].

On the other hand, mechanical (physics-based) modelling has demonstrated to be very useful to develop knowledge about relations between structure and mechanical properties, by subjecting a virtual food to a virtual standard mechanical test or a virtual oral breakdown. With the aid of imaging methods such as X-ray microtomography (XMT or micro-CT), realistic 3D geometric models can be obtained for simulation purposes [8]. Besides large deformation analysis, fracture mechanics has been applied to understand the breakage of solids under large deformation; this physics-based approach allows determining intrinsic properties of food materials, which do not depend on test parameters or sample geometry, e.g., Young’s modulus, fracture stress, fracture toughness, and the critical stress intensity factor [18]. For instance, different authors have employed the finite element method (FEM) to model mechanical tests such as compression, in order to obtain mechanical properties of materials, e.g., References [62,174,175,176,177]. Due to limitations of FEM to simulate large deformation and fragmentation behavior, some authors applied meshfree methods. For example, Harrison et al. [178] developed a coupled biomechanical-smoothed particle hydrodynamics (SPH) model of human mastication, in order to predict the mechanical behavior and breakdown of two agar model foods. The authors reported that a further step would be to extend this model for predicting flavor release during oral processing from mechanical properties. Likewise, Hedjazi et al. [179] studied the fragmentation behavior of breakfast cereals by using the discrete element method (DEM).

In summary, modelling approaches, especially physics-based models, in combination with advanced numerical methods and powerful imaging techniques, certainly help to increase the understanding about the relationships between structure, mechanical properties, and oral processing, in order to design foods with specific characteristics. For instance, structure can affect the digestion process [61], so such understanding is crucial to deliver healthier food products. In this regard, it is worth making a final comment about additive manufacturing (AM) or 3D printing, which has emerged as a promising technique for food tailoring or customization [180]. AM can be used to perform reverse engineering, i.e., to utilize knowledge and information about products/processes to improve or reuse the (direct) engineering process for delivering a new product. For example, topology optimization (optimal design of geometry) is a reverse engineering tool that can be used in AM. So, virtual design and reverse engineering can produce tailor-made foods for different objectives and functionalities, e.g., specific fragmentation performance, controlled released of active compounds, etc. [181]. Some recent works have been dedicated to study mechanical behavior of 3D-printed foods. For instance, Jonkers et al. [182] proposed a constitutive model (elasto-viscoelastic) to describe large deformation behavior of 3D-printed starch-based foods. In an interesting article, Piovesan et al. [183] applied a computer aided engineering (CAE) methodology to design 3D-printed foods with tunable mechanical properties, by using Young’s modulus as texture descriptor. Finite element modelling was used to analyze the relationship between Young’s modulus of 3D-printed cookies with a honeycomb structure and their parameters. The authors reported that wall thickness and cell size can be used as design parameters to customize texture based on Young’s modulus. For sure, more works will be dedicated to AM and mechanical modelling in the near future, to further elucidate the relations between food structure and oral processing, considering the ultimate challenge of optimal design of foods.

## 4. Conclusions

A wide variety of modelling approaches have been applied to describe and predict volume change and large deformation of food materials in various processes. A classification of such models was proposed in this work, based on the prediction capability and hypotheses of each approach: (i) Simple models are able to predict overall volume change and related properties (e.g., bulk porosity), either from empirical data or from theoretical simplifications; (ii) physics-based models can predict local deformation and porosity evolution, and stress field and related magnitudes via mechanical modelling (mechanics-based models). In some cases, mechanical modelling is avoided by using theoretical assumptions or semi-empirical approaches to compute solid velocity in a simplified way (hybrid models). Certainly, mechanics-based modelling presents relevant advantages in terms of prediction capability and interpretation of involved phenomena, providing useful tools and insights for a better understanding of the relationships between composition and structure of raw materials, processing conditions, and properties of final products. Besides macroscale modelling framework, which is commonly applied in food engineering, microscale and multiscale approaches have been utilized, as well as meshfree methods and well-suited concepts and theories from other fields, e.g., soft condensed matter and polymer science. These “novel” perspectives and methods will surely improve physics-based models in the food engineering field.

On the other hand, there are still some bottlenecks to be addressed in order to further expand the development and application of physics-based models. One major bottleneck involves the availability of appropriate thermophysical and mechanical properties, considering the actual composition and structure of food materials, and realistic processing conditions. In this regard, the influence of state transitions and anisotropy effects need to be incorporated. It has been extensively reported the key role of water as plasticizer and as agent for deformation, as well as the importance of structural arrangement of materials to transport phenomena. Another aspect to improve is the validation of simulation results, which in many cases is performed by using average or overall values, partially due to experimental limitations for the acquisition of detailed and adequate data. A full and direct validation of models will consolidate their predictive potential. The third issue concerns the need for the development of modelling frameworks and customized modules in simulation software, with focus on food engineering applications. This will certainly help to expand the application of physics-based models, including education and training aspects of (nonlinear) solid mechanics, considering that implementation of these models is not straightforward.

Towards the development of a food engineering-oriented physics-based modelling framework, different products/processes can be grouped or classified according to the main mechanism or driving force causing large deformation and volume change: (i) Moisture-driven deformation in drying, hydration, and cooking; (ii) pressure-driven deformation in baking, extrusion, and puffing; and (iii) mechanical-driven deformation in oral processing. In addition, other driving forces can be relevant and thus need to be considered and coupled accordingly, e.g., temperature can affect deformation by modifying the state and, thus, the mechanical properties of the material. Besides, this classification can be useful to identify and solve common problems in various products/processes, and to increase feedback between different research areas.

Finally, it is worth mentioning the need for bridging the gap between process modelling efforts (*prediction of* temperature, moisture content, deformation, etc.) and product-focused works (*impact of* processing on quality and sensory aspects). That is, the gap between “*prediction of*” and “*impact of*” works. An example of this gap is the lack of more articles dealing with the prediction of food texture from physics-based models. Certainly, mechanical modelling can provide useful tools for this aim. More efforts are required in this direction, towards the development of mechanistic digital twins and optimal design of food products/processes.

## Figures and Tables

**Table 1:** Summary of applications discussed in Section 3, as a reference guide.

Process	Product(s)	Type(s) of Deformation	Driving Force(s) for Deformation	Recommended References
Drying	Fruits and vegetables (also mushrooms and meat products)	Shrinkage	Dehydration causing loss of turgor pressure in cells	[20,36,37,38,39]
Hydration (or soaking)	Grains (legumes and cereals)	Swelling	Water absorption by biopolymers	[40,41,42,43,44]
Cooking	Meat products	Shrinkage	Proteins denaturation increasing the swelling pressure	[9,45,46,47,48]
Baking	Bread, cakes	Expansion	Gas pressure rise inside of pores of dough	[49,50,51,52,53]
Extrusion and Puffing	Snacks, breakfast cereals	Expansion	Pressure rise due to water vaporization	[54,55,56,57,58]
Oral processing	Solid foods	Compression and fracture	Mechanical stress	[18,59,60,61,62]
